# GABAergic Gene Regulatory Elements Used in Adeno-Associated Viral Vectors

**DOI:** 10.3389/fneur.2021.745159

**Published:** 2021-10-04

**Authors:** Robert Duba-Kiss, Yosuke Niibori, David R. Hampson

**Affiliations:** ^1^Department of Pharmacology and Toxicology, Faculty of Medicine, University of Toronto, Toronto, ON, Canada; ^2^Department of Pharmaceutical Sciences, Leslie Dan Faculty of Pharmacy, University of Toronto, Toronto, ON, Canada

**Keywords:** enhancer, GABA, gene therapy, interneuron, promoter, viral vector

## Abstract

Several neurological and psychiatric disorders have been associated with impairments in GABAergic inhibitory neurons in the brain. Thus, in the current era of accelerated development of molecular medicine and biologically-based drugs, there is a need to identify gene regulatory sequences that can be utilized for selectively manipulating the expression of nucleic acids and proteins in GABAergic neurons. This is particularly important for the use of viral vectors in gene therapy. In this Mini Review, we discuss the use of various gene regulatory elements for targeting GABAergic neurons, with an emphasis on adeno-associated viral vectors, the most widely used class of viral vectors for treating brain diseases.

## Adeno-Associated Viruses Used in Gene Therapy

Gene therapy based on the delivery of therapeutic transgenes to affected tissues by recombinant adeno-associated viruses (rAAVs) has been intensively studied in preclinical and clinical research. AAVs are small viruses (25 nm particle diameter) that exhibit several properties that make them attractive for therapeutic applications. They are non-pathogenic in humans and other mammals and elicit mild to moderate immune responses. rAAV genomes are maintained in an extrachromosomal state (1) and generally do not recombine into the host genome, minimizing the risk of insertion-based genotoxicity. Despite existing outside of the host genome, rAAV DNA persists in non-dividing host cells, such as most neurons, and mediates transgene expression over a protracted time frame – studies have demonstrated expression 8 and 15 years after treatment in the brains of non-human primates (2, 3).

Several natural AAV serotypes exist, each of which display different levels of tropism for different cells and tissues. Of these AAV serotypes 2, 5, 8, and 9 have shown the highest tropism in the central nervous system (CNS), and thus have been most broadly applied for CNS delivery (4). In addition, artificial serotypes have been created that convey more favorable properties, such as rAAV-PHP.B, which was designed to efficiently cross the blood-brain barrier after intravenous injection (5). However, the ability of various rAAV serotypes to selectively target individual cell types is currently still limited. Instead, cell-type specific promoter or enhancer gene regulatory sequences can be utilized to direct transgene expression to selected cell types.

The wild type AAV genome is single stranded in structure, consists of two genes encoding the capsid and reproduction proteins, and is flanked by inverted terminal repeats (6). rAAVs are constructed by removal of the native viral genome and insertion of the recombinant gene of interest and associated gene regulatory elements, along with two essential flanking inverted terminal repeats (7). A drawback of rAAVs is that the maximum size of the DNA they can carry is limited to the size of the native genome: ~4.8 kilobases (kb) for single stranded rAAVs, and ~2.4 kb for double stranded self-complementary rAAVs. This limited space must include the two inverted terminal repeat sequences, a promoter and/or other regulatory elements, and the polyadenylation region, and therefore restricts the size of the transgene open reading frame that can be packaged within an AAV vector. For example, rAAVs that utilize regulatory elements that are 2,000 bases in length would limit the inserted coding sequence for the transgene, polyadenylation sequence, and inverted terminal repeats to about 2,800 bases in single stranded rAAV vectors and only about 400 bases in self-complimentary rAAV vectors.

In some cases, the limited DNA packaging capacity of these vectors presents a challenge for the creation of therapeutic rAAVs. Certain disorders involve mutations in genes larger than the rAAV packaging limit, rendering a direct gene replacement strategy infeasible. Depending on the mechanism underlying the disorder, therapeutic strategies using smaller, alternate genes may be an option. Of course, the size of a gene that can be packaged in an rAAV can be increased by minimizing the size of promoter and other regulatory elements used to drive transgene expression. Thus, the creation of miniaturized promoter and enhancer elements that maintain cellular specificity and the ability to drive robust expression is an active area of research. A list of studies using GABA gene-based regulatory elements is shown in Table 1.

**Table 1:** GABAergic neuron-selective regulatory elements tested with rAAVs.

**Promoter/enhancer**	**Associated gene**	**Element size**	**Injected animal**	**Injection route**	**Serotype**	**Expressed protein**	**Time to collection**	**Analysis technique**	**Brain region analyzed**	**Marker for GABAergic neurons**	**Specificity for marker**	**Ref**
**PROMOTER SEQUENCES**												
**Gad1 Promoter**	*Gad1*	2700 bp	Neonatal Mouse	ICV + ICM	9	NaVβ1-c- myc	15–16 days	IHC	Frontal Cortex	Anti-GABA	30%	(8)
									Visual Cortex Hypothalamus	Anti-GABA Anti-GABA	14% 28%	
							2–3 months	IHC	Frontal Cortex	Anti-GABA	9%	
									Visual Cortex Hypothalamus	Anti-GABA Anti-GABA	13% 17%	
	*GAD1*	3090 bp	Mouse (Age not Specified)	IC	9	YFP	2 weeks	IHC	Cerebral Cortex	Anti-GABA	Data not available	(9)
**mGad65**	*Gad2*	2540 bp	Adult Mouse	IV	PHP.B	GFP	3 weeks	IHC	Motor Cortex	VGAT-tdTomato Mouse	96%	(10)
**ENHANCER ELEMENTS**												
**I12b**	*dlx1/2*	450 bp	Adult Mouse	IC	5	eYFP	7–8 weeks	IHC	Medial Prefrontal Cortex	I12b-Cre Mouse (GABA)	90%	(11)
**I56i**	*dlx5/6*	400 bp	Adult Mouse	IV	PHP.B	GFP	3 weeks	IHC	Motor Cortex	VGAT-tdTomato Mouse	88%	(10)
				IC	N/A	GFP	7 days	IHC	Somatosensory Cortex	Dlx6a-Cre Mouse (GABA)	93%	(12)
				IH	N/A	GFP	7 days	IHC	Hippocampus - CA1	Dlx6a-Cre Mouse (GABA)	92%	
			Adult Zebra Finch	Intra-HVC	N/A	GFP	2–4 weeks	IHC	HVC	Anti-GABA	91%	
			Adult Gerbil	IC	N/A	mCherry	2-4 weeks	IHC	Auditory Cortex	Anti-GAD67	94%	
			Juvenile Ferret	IC	N/A	GFP	2 weeks	IHC	Visual Cortex	Anti-GAD67	98%	
			Adult Marmoset	IC	N/A	GFP	3 months	IHC	Visual Cortex	Anti-GAD67	93%	
			Neonatal mouse	ICV	9	tdTomato	5 weeks	IHC	Cortex	Gad67-GFP Mouse	85%	(13)
									Hippocampus		85%	
**h12R**	*DLX1/2*	380 bp	Adult Mouse	IC IH	2/7 2/7	tdTomato tdTomato	10–15 days 10–15 days	ISH ISH	Cerebral Cortex Hippocampus	GAD65 Probe GAD65 Probe	93% 96%	(14)
**h56D**	*DLX5/6*	840 bp	Adult Mouse Juvenile Gerbil Adult Marmoset	IC IH IC IH IC	2/7 2/7 2/7 2/7 2/7	tdTomato tdTomato tdTomato tdTomato tdTomato	10–15 days 10–15 days 10–15 days 10–15 days 4–5 weeks	ISH ISH ISH ISH ISH	Cerebral Cortex Hippocampus Cerebral Cortex Hippocampus Cerebral Cortex	GAD65 Probe GAD65 Probe GAD65 Probe GAD65 Probe GAD65 Probe	93% 95% 84% 98% 97%	(14)
**E2**	*Scn1a*	620 bp	Adult Mouse Adult Rat Adult Macaque Adult Marmoset	IV IC IC IV	PHP.eB 1 PHP.eB 9	dTomato eYFP dTomato dTomato	3 weeks 2–8 weeks 2–8 weeks 2–8 weeks	ISH IHC IHC IHC IHC	Somatosensory Cortex Somatosensory Cortex Cerebral Cortex Cerebral Cortex Cerebral Cortex	GAD67 Probe Anti-PV Anti-PV Anti-PV Anti-PV	98% 74% 93% 87% 92%	(15)
**E11**	*Pvalb*	500 bp	Adult Mouse	IV	PHP.eB	GFP	3 weeks	IHC	Somatosensory Cortex	Anti-GAD67 Anti-PV	95% 91%	(15)
**E14**	*Acan*	500 bp	Adult Mouse	IV	PHP.eB	GFP	3 weeks	IHC	Somatosensory Cortex	Anti-GAD67 Anti-PV	93% 94%	(15)
**E22**	*Tmem132c*	440 bp	Adult Mouse Adult Macaque	IV IC	PHP.eB PHP.eB	GFP GFP	3 weeks 8 weeks	IHC IHC	Somatosensory Cortex Cerebral Cortex	Anti-GAD67 Anti-PV Anti-PV	98% 93% 92%	(15)
**E29**	*Inpp5j*	630 bp	Adult Mouse Adult Macaque	IV IC	PHP.eB PHP.eB	GFP GFP	3 weeks 8 weeks	IHC IHC	Somatosensory Cortex Cerebral Cortex	Anti-GAD67 Anti-PV Anti-PV	96% 94% 81%	(15)
**eHGT_079h**	*TAC1*	600 bp	Juvenile-Adult Mouse	IV	PHP.eB	SYFP2	21–28 days	mFISH	Cerebral Cortex	PV Probe	87%	(16)
			Adult Macaque	IC	PHP.eB	SYFP2	51–113 days	IHC	Occipital Cortex	Anti-PV	86%	
												
**eHGT_082h**	*NOS1AP*	700 bp	Juvenile-Adult Mouse	IV	PHP.eB	SYFP2	21–28 days	mFISH	Cerebral Cortex	PV Probe	93%	(16)
			Adult Macaque	IC	PHP.eB	SYFP2	51–113 days	IHC	Occipital Cortex	Anti-PV	98%	
**eHGT_128h**	*VAV3*	430 bp	Juvenile-Adult Mouse	IV	PHP.eB	SYFP2	21–28 days	mFISH	Cerebral Cortex	PV Probe	93%	(16)
			Adult Macaque	IC	PHP.eB	SYFP2	51–113 days	IHC	Occipital Cortex	Anti-PV	95%	
**eHGT_140h**	*NRF1*	300 bp	Juvenile- Adult Mouse	IC	PHP.eB	SYFP2	21–28 days	mFISH	Cerebral Cortex	PV Probe	99%	(16)
			Adult Macaque	IC	PHP.eB	SYFP2	51–113 days	IHC	Occipital Cortex	Anti-PV	95%	
									Temporal Cortex	Anti-PV	77%	
									Somatosensory Cortex	Anti-PV	92%	
									Motor Cortex	Anti-PV	88%	

*Acan, aggrecan gene; bp, base pairs; dlx, distal-less homeodomain transcription factor; eYFP, enhanced yellow fluorescent protein; GABA, gamma aminobutyric acid; GAD65, glutamate decarboxylase 65; GAD67, glutamate decarboxylase 67; GFP, green fluorescent protein; IC, intra-cortical injection; ICM, intra-cisternal magna injection; ICV, intra-cerebroventricular injection; IH, intra-hippocampal injection; IHC, immunohistochemistry; Inpp5j, inositol polyphosphate-5-phosphatase J gene; ISH, in situ hybridization; IV, intra-venous injection; mFISH, multiplexed fluorescence in situ hybridization; NOS1AP, nitric oxide synthase 1 adaptor protein gene; NRF1, nuclear respiratory factor 1 gene; PV, parvalbumin; Pvalb, parvalbumin gene; Scn1a, sodium voltage-gated channel alpha subunit 1 gene; SYFP2, strongly enhanced yellow fluorescent protein 2; Tac1, tachykinin precursor 1 gene; Tmem132c, transmembrane protein 132C gene; VAV3, Vav guanine nucleotide exchange factor 3 gene; VGAT, vesicular gamma-amino acid transporter; YFP, yellow fluorescent protein*.

## GABAergic Genes and GABA-Related Genetic Disorders

Brain cells can be broadly categorized into excitatory and inhibitory neurons, and glial cells such as astrocytes, oligodendrocytes, and microglia. Inhibitory neurons, also called GABAergic neurons that produce the inhibitory neurotransmitter gamma-aminobutyric acid (GABA), account for about 10–20 % of the total neuronal population in the mouse cerebral cortex (17). GABAergic neurons are required to maintain the balance between excitation and inhibition and the control of physiological activity in the brain (18).

Examples of genes that regulate GABAergic neuronal activity and are expressed specifically in GABAergic neurons include the *GAD1, GAD2*, and *SLC32A1* genes. *GAD1* and *GAD2* encode the decarboxylase enzymes, GAD67 and GAD65, respectively, which catalyze the synthesis of GABA from L-glutamate. *SLC32A1* encodes a vesicular GABA transporter (VGAT) that concentrates GABA into the synaptic vesicles. GABAergic neurons can also be divided into distinct subclasses such as those expressing the genes encoding the Ca^2+^-binding protein parvalbumin (*PVALB*), the neuropeptide somatostatin (*SST*), vasoactive intestinal peptide (*VIP*), and the ionotropic 5-hyroxytryptamine 3a serotonin receptor (*HTR3A*).

Impairment of GABA synthesis or GABA neuron activity is thought to lead to neurodevelopmental disorders, epilepsy, and psychiatric disorders. For instance, GABA synthesis failure by bi-allelic loss-of-function mutations in the *GAD1* gene causes early infantile epilepsy, severe cleft palate, and neonatal death (19, 20). In addition, reduced GABAergic neuronal activity, abnormal brain activity, or GABA markers in postmortem studies have been observed in epilepsy (21), autism (22, 23), schizophrenia (24), Alzheimer's disease (25), Down's Syndrome (26), and bipolar disorder (27). Results from postmortem brain analyses of schizophrenia subjects have demonstrated decreased expression of GAD67 in the prefrontal cortex (28–30). Dravet syndrome, a genetic disorder which causes childhood epilepsy, occurs from mutations in the *SCN1A* gene encoding the Nav1.1 voltage-gated sodium channel. Experiments in mice have demonstrated that Nav1.1 is highly expressed in GABAergic neurons, and mice with mutations in *Scn1a* have an impaired ability to maintain the normal high frequency firing rate in inhibitory neurons (31, 32). Thus, overall, there is a need to develop an expanded toolbox of GABA-related gene regulatory elements for use in viral vectors. It should be noted that most of the constructs described in this review have only been analyzed using a single study protocol, and that their efficacies may vary under different experimental conditions.

## The *DLX* Gene Enhancer Elements

The distal-less homeodomain transcription factor (*DLX*) family is a gene family highly conserved across vertebrates. *DLX 1, 2, 5*, and *6* are expressed in the CNS during brain development and promote the expansion and differentiation of GABAergic neurons. *DLX1* and *DLX2* are important in the migration of nascent cortical GABAergic neurons and in the embryonic expression of GABAergic neuron-specific genes such as *GAD1, GAD2*, and *SLC32A1* (33, 34). The precise roles of *DLX5* and *DLX6* are less clear although they are known to be important for the differentiation and maturation of cortical parvalbumin (PV) GABAergic neurons (35). Similarly, *DLX1* is important for the development of cortical SST, calretinin, and neuropeptide Y GABAergic neurons (36). Expression of the DLX proteins declines after the neonatal period, although they are still present at lower levels in the CNS during adulthood (37, 38).

The *DLX* genes are grouped into bicistronic gene clusters and are regulated by intergenic enhancer sequences with high degrees of homology between mice and humans. *Dlx1/2* and *Dlx5/6* each form such bicistronic clusters, and in mice are regulated by the enhancer domains I12b (39) and I56i (12, 40), respectively. Analogous enhancer sequences are found in human *DLX* genes: h12R (found in *DLX1/2*) and h56D (found in *DLX5/6*) (14). Each of these enhancers, when packaged with a minimal promoter onto rAAV vectors, effectively direct transgene expression to GABAergic neurons. Direct injection of rAAVs carrying I56i, h12R, or h56D into the murine cerebral cortex and hippocampus resulted in the transduction of 80–90% of cortical and hippocampal GABAergic neurons after each injection, respectively (12, 14). Intravenous injection of rAAV-I56i labeled 57% of murine cortical GABAergic neurons (10). All of the aforementioned *DLX* enhancers (I12b, I56i, h12R, and h56D) directed transgene expression to murine cortical and hippocampal GABAergic neurons with approximately 90% or greater specificity [(10–12, 14); see Table 1]. Similar levels of GABAergic neuronal specificity and coverage were observed in gerbils and in non-human primates (12, 14). Moreover, application of an rAAV-I56i, as well as a rAAV with a triplicate repeat of an I56i core sequence (rAAV-DLX2.0), to *ex vivo* human cortical tissue demonstrated that these vectors effectively transduced human GABAergic neurons (16). A rAAV using a mouse *dlx5/6* enhancer was also used in Scn1a mutant mice, a mouse model of Dravet Syndrome. Neonatal injections into the ventricles resulted in transduction of about 20% of cortical GABAergic interneurons near the injection site, and caused normalization of GABA neuron firing and a reduction in febrile seizures (13).

Despite the excellent ability of these enhancers to target GABAergic neurons in anterior brain regions, neither intravenous injection of rAAV-I56i (10, 16), nor intra-inferior collicular injection of rAAV-h65D (14) induced transgene expression in posterior brain regions (such as the midbrain, cerebellum, and brainstem). The non-functionality of *DLX* enhancers in the posterior brain poses a limitation for studies of candidate therapies requiring posterior brain or CNS-wide GABAergic neuronal targeting; other regulatory elements will likely be necessary in these cases.

## The *GAD1* Promoter

The *GAD1* and *GAD2* genes are evolutionarily conserved across eukaryotes. In the human cerebral cortex, expression of both *GAD1* and *GAD2* rapidly increases after birth; *GAD1* continues this upward trajectory for another 2–3 years after birth, while *GAD2* reaches a plateau around 1 year after birth (41). In mice, deletion of *Gad1* caused dramatically reduced GABA levels in the CNS, severe cleft palate, respiratory failure, and death shortly after birth (42). The expression of *GAD1* is regulated by homeobox domain transcription factors DLX1 and DLX2. Multiple putative binding sites for DLX proteins have been identified in the 5' intergenic region of the *Gad1* gene (34). In addition, the expression levels of GAD67 were decreased in *Dlx1/2* knockout mice, suggesting that *Dlx1/2* regulates *Gad1* transcription (34, 43).

The 5' intergenic region between the *GAD1* gene and the 5' upstream gene *ERICH2*, spans 23 kb. DNA sequences from a segment of the 5' intergenic region and exon 1 containing the transcription start site of *GAD1* have been utilized in rAAVs (8, 9). Niibori et al. reported that an rAAV containing a 2.7 kb section of the mouse *Gad1* promoter showed 80% overall neuronal specificity and 12–30% GABAergic neuronal selectivity, depending on the brain region examined; AAV-mediated transgene expression was found in the forebrain and midbrain, but not in the cerebellum or brainstem (8). By comparison, Liu et al. (9) reported that a 3.1 kb section of the human *GAD1* promoter showed complete GABAergic neuronal specificity; however, no data were presented to support this statement. The difference in GABA specificity reported by Niibori et al. (8) and Liu et al. (9) could be due to the absence of a GABA specificity element in the shorter 2.7 kb construct vs. the longer 3.1 kb construct. However, it is also possible that differences in the experimental conditions of the two studies (i.e., age, route of injection, and dose of rAAV), rather than differences in DNA sequences, contributed to the difference in GABAergic neuronal specificity. For example, lowering the dose of an rAAV encoding a neuron-specific (Synapsin) promoter markedly increased its specificity for GABAergic neurons (44). Additionally, the GABA selectivity of rAAVs injected during the early postnatal period was reported to be lower than that of rAAVs injected into adult mice (15).

## The *GAD2* Promoter

Like *GAD1, GAD2* expression begins during embryonic development and is regulated by the transcription factors DLX1 and DLX2. Unlike *GAD1*, the null mutation of *GAD2* is not fatal, although *Gad2*^−/−^ mice show increased fearful and anxious behavior, as well as elevated susceptibility to seizures (45, 46).

An rAAV carrying a 3 kb region of the murine *Gad2* promoter (3,000 base pairs upstream from the start codon) was used to examine enhanced green fluorescent protein (eGFP) transgene expression in mouse brain; this rAAV displayed 20% specificity to excitatory neurons in the murine cerebral cortex (10). However, another construct carrying a deletion of *Gad2* exon 1 abolished excitatory neuron expression and induced labeling of 60% of cortical GABAergic neurons with 96% GABA neuron specificity after intravenous injection of AAV-PHP.B (10). rAAVs carrying this truncated *Gad2* promoter (denoted as rAAV-mGad65 by the authors of the study) showed broad expression in the mouse CNS, including in anterior areas such as the cerebral cortex, hippocampus, and striatum, and in posterior areas including the midbrain, cerebellum, and brainstem. In the cerebellum, this construct induced high transduction of eGFP in GABAergic neurons in the molecular layer (coverage = 80%), but low transduction of GABAergic Golgi cells in the granule layer (coverage = 8%) and of GABAergic Purkinje cells (coverage = 1%). This rAAV construct is the first to use sequences from the *Gad2* promoter to direct rAAV-mediated transgene expression to GABAergic neurons. Despite limited research with *Gad2* sequences, the broad CNS distribution of expression and high degree of specificity to GABAergic neurons demonstrates the potential utility in gene therapy. Additional testing in non-human primates would be useful to evaluate whether such *Gad2* promoter constructs might be useful for clinical applications.

## The VGAT Promoter

The vesicular gamma-amino acid transporter VGAT, previously known as the vesicular inhibitory amino acid transporter, is coded for by the *SLC32A1* gene. Analysis of the mouse *Slc32a1* promoter indicated that a minimal promoter region containing a CG rich sequence is sufficient for efficient expression in neural stem and precursor cells (47, 48). Deletion of this CG rich region greatly reduced its activity in neural precursor cells. It was suggested that the CG rich region may be acting as a core promoter element, mediating the activity of SP1 or other SP family transcription factors (47, 48).

To date, no studies have reported the use of the *Slc32a1* promoter region in rAAV gene therapy experiments. However, a cell type-specific promoter-driven fluorescent reporter construct was developed that utilized the human vesicular GABA transporter *SLC32A1* promoter to drive the expression of mCherry specifically in *Vgat-*expressing neurons. A 1,865 bp region of the *SLC32A1* gene, including 262 bp downstream of the transcription start site and 1,603 bp upstream of the transcription start site, was PCR amplified from genomic DNA. This region overlaps with peaks for several markers of promoter activation, histone H3K4 monomethylation (H3K4me1), histone H3K4 trimethylation H3K4me3, and RNA polymerase II (Pol2) binding (49). The transduction of iPSC derived forebrain neuronal cultures with a hVGAT promoter-mCherry lentiviral reporter construct specifically labeled GABAergic neurons. Immunocytochemical analysis of hVGAT-mCherry expression showed prominant co-labeling with GABAergic neuronal markers including VGAT, GABA, and GAD67 (49).

## The Somatostatin Promoter

SST is a peptide hormone that regulates the endocrine system, functions as a neuropeptide, and affects cellular proliferation. In the brain, SST is selectively expressed in a subset of GABAergic interneurons. Impaired function of this class of interneurons has been suggested to be involved in the pathophysiology of depression, bipolar disorder, and schizophrenia (50). Promoter sequences derived from putative orthologous fugu (Pufferfish) *Sst* have been generated and tested, as were composite *Sst* regulatory elements containing transcription factor binding sites (51). A 2,597 base pair fragment of the fugu *Sst* promoter was packaged into a recombinant rAAV2/1 (AAV2 backbone packaged with AAV1 capsid) in front of an eGFP reporter, and injections were made into the somatosensory cortex of adult mice. In the somatosensory cortex this rAAV displayed 55–88% coverage across subclasses of inhibitory neurons including SST, PV, neuropeptide Y, and VIP-positive neurons. In contrast, an attempt to use a segment containing the mouse *SST* promoter in a lentivirus construct was not successful (51).

An intersection strategy was used in rAAV vector design by Mehta et al. whereby rAAV *Sst*-Cre recombinase and a construct with *DLX* enhancers and eGFP with a double-floxed inverted open reading frame, were co-injected into the hippocampus of adult mice; eGFP was expressed only when both promoters were active in the same cell (14). The selected DNA sequence included a segment that was conserved between the mouse and human genes, extended 2,000 bases upstream of the mouse Sst start codon, and encompassed three conserved domains. This arrangement resulted in high GABA interneuron specificity and coverage in the hippocampus (about 90% for both).

## Regulatory Elements in the Vasointestinal Peptide and HTR3A Genes

The *VIP* gene encoding the neurotransmitter vasointestinal peptide (VIP) is expressed in GABAergic neurons, especially in cell populations distinct from PV and SST expressing neurons (52). VIP is a neurotransmitter expressed in forebrain regions such as the amygdala, and in the midbrain including the suprachiasmatic nucleus (52, 53). The VIP pathway is associated with regulation of appetite and circadian rhythm (54, 55). The *HTR3A* gene encodes the 5-hydroxytryptamine 3A serotonin receptor (5HT3AR). Abnormalities in the 5HT3AR pathway have been associated with psychiatric disorders such as bipolar disorder, schizophrenia (56), and autism (57). In mice, *Htr3a* is also expressed primarily in GABAergic neurons distinct from *Pvalb* and *Sst* expressing neurons, and is not expressed in excitatory neurons or glial cells (52, 58). *Vip* is expressed in 40% of *Htr3a* expressing GABAergic neurons (58, 59). To date there have been no reports of development of rAAV or other viral vectors using the *Htr3a* or *Vip* promoters. However, identified functional promoters/enhancers in *Vip*-expressing neurons include *Dlx* enhancers from human and mouse (12, 14), mouse *Scn1a* enhancers (15), and *Pvalb, Sst*, and, *Npy* promoters from pufferfish (51).

## The Parvalbumin Promoter and Expression in Parvalbumin Neurons

PV is a small, Ca^2+^-binding protein that is prominently expressed in a subset of GABAergic neurons throughout the CNS. Examples include cortical basket and chandelier cells (60), some hippocampal interneurons (61), and interneurons in the reticular thalamic nucleus (62). Dysfunction of PV-expressing GABAergic neurons have been implicated in several neurological and psychiatric disorders such as epilepsy (63, 64), autism (65–67), and schizophrenia (30, 68, 69). Thus, successful targeting of PV GABAergic neurons with rAAVs could have implications for gene therapy treatments of these diseases.

A region of the *PVALB* promoter with high homology between humans and mice was shown to be unable to restrict rAAV-mediated expression to PV GABAergic neurons (although the exact sequence and position of the sequence used was not reported in the study) (14). However, another study using a lentiviral vector and segments of the *Pvalb* promoter (−4 to −1,880 and −4 to −780) conserved between the mouse and the macaque, reported high specificity to PV GABAergic neurons after injection into the thalamic reticular nucleus of adult mice (70). However, these *Pvalb* promoter regions have not been tested in rAAVs.

Another method to restrict rAAV transgene expression to PV GABAergic neurons in mice is the intersectional strategy by using two rAAV constructs, one carrying the Cre-recombinase gene, and the other carrying a Cre-dependent reporter transgene (see the somatostain promoter section above). The Cre-recombinase-carrying rAAV used a promoter from the *PaqR4* gene, which is active in PV GABAergic neurons, but also in non-GABAergic cells, and the reporter-carrying rAAV contained the h56D enhancer which expresses in GABAergic neurons. Reporter gene expression was therefore only induced in PV GABAergic neurons where both rAAVs were present (80% and 69% GABA specificity in mouse hippocampus and cortex, respectively) (14).

Numerous short, conserved enhancer elements have been identified by chromatin accessibility profiling that effectively restrict rAAV-mediated expression to PV GABAergic neurons (15, 16). These enhancers directed rAAV-mediated expression to cortical PV GABAergic neurons with 85–90% specificity after intravenous injection into mice, but varied with respect to the degree of distribution of transgene expression they induce in the murine CNS. Notably, an enhancer from the *Inpp5j* gene drives particularly robust and widespread CNS expression (transducing the cerebral cortex, hippocampus, striatum and cerebellum) (15). Another enhancer from the *NOS1AP* gene is particularly effective at transducing posterior regions such as the midbrain, brainstem and cerebellar nuclei, but shows comparatively limited expression in the forebrain (16). These PV GABAergic neuron-selective enhancers are also operative in non-human primates; direct intra-cortical injection of macaques with enhancer-carrying rAAVs directed reporter expression to cortical PV GABAergic neurons with 80% or greater specificity (15, 16). The PV GABAergic neuron selective enhancers that showed the highest specificity are listed in Table 1. The excellent PV GABAergic neuron targeting ability of these enhancer elements, as well as their small size and utility in non-human primates, make them attractive candidates for potential rAAV therapies.

## Summary

Perturbations in GABAergic neurons cause an imbalance between excitatory and inhibitory brain activity and can lead to CNS disease. GABAergic neuron-specific rAAVs are expected to drive therapeutic protein expression in GABA neurons and improve symptoms by normalization of GABAergic neuronal function. The possibility exists that certain disorders may alter the transcriptional activities of GABAergic neurons and render promoter/enhancer elements that function in healthy neurons ineffective. Thus, when developing a candidate therapy, it will be important to evaluate the GABAergic neuronal targeting ability of the rAAV construct in the pertinent disease model to ensure its functionality is not altered. Although the discovery of GABA-based regulatory elements for use in rAAVs is in an early stage of development, the various GABA promoters and enhancers studied so far varied in length from about 300 bases to 2.5 kb and displayed a range of GABAergic neuronal specificities (about 30–90%). Among them, a fragment of the mouse *Gad2* promoter appeared to operate in multiple GABA neuron subtypes and across many regions throughout the CNS (10). However, the majority of studies reported to date were based on the analysis of fluorescent reporter proteins and did not assess therapeutic efficacy in disease models. We expect this state-of-the-art to improve rapidly whereby GABA neuron-directed viral vectors will be tested in additional animal models of neurological and psychiatric disorders, with the expectation that some of these candidate gene regulatory elements in combination with therapeutic transgenes will advance to clinical testing.

## Author Contributions

All authors listed have made a substantial, direct and intellectual contribution to the work, and approved it for publication.

## Conflict of Interest

The authors declare that this study received funding from Regenxbio Inc. The funder had the following involvement in the study: study design, decision to publish, and preparation of the manuscript.

## Publisher's Note

All claims expressed in this article are solely those of the authors and do not necessarily represent those of their affiliated organizations, or those of the publisher, the editors and the reviewers. Any product that may be evaluated in this article, or claim that may be made by its manufacturer, is not guaranteed or endorsed by the publisher.
